# Insecticidal efficacy against *Phlebotomus perniciosus* in dogs treated orally with fluralaner in two different parallel-group, negative-control, random and masked trials

**DOI:** 10.1186/s13071-021-05128-z

**Published:** 2022-01-08

**Authors:** Gioia Bongiorno, Leon Meyer, Alec Evans, Nouha Lekouch, Padraig Doherty, Rafael Chiummo, Luigi Gradoni

**Affiliations:** 1 Unit of Vector-Borne Diseases, Istituto Superiore Di Sanità, 00161 Rome, Italy; 2 Clinvet SA, Mohammedia, 28815 Morocco; 3 Iorras Product Development Limited, Glenamoy–Ballina, Co. Mayo F26Y286 Ireland; 4 MSD Animal Health Innovation GmbH, 55270 Schwabenheim an der Selz, Germany

**Keywords:** Dog, Fluralaner, Insecticidal efficacy, *Phlebotomus perniciosus*

## Abstract

**Background:**

Dogs are the reservoir host of *Leishmania infantum*, the agent of zoonotic visceral leishmaniasis (VL), which is transmitted by the bite of phlebotomine sand flies. The sand fly *Phlebotomus perniciosus* is the main vector of zoonotic VL in the western Mediterranean region. Fluralaner has been shown to effectively kill this vector. The aim of this study was to evaluate the insecticidal efficacy of oral fluralaner in dogs bitten by *P*. *perniciosus*.

**Methods:**

Two parallel-group, negative-controlled, randomized, masked laboratory trials with equivalent designs were performed in two different locations using two different pathogen-free laboratory-bred *P. perniciosus* strains for the challenge. In each trial, 12 purpose-bred beagles, initially ranked on natural attractiveness to sand flies, were randomly allocated to two groups (6 animals/group). Dogs in one group received fluralaner orally at the approved dose on day 0, and dogs in the control group were not treated. Each dog was subsequently exposed to an average of 70 unfed live sand fly females on days 1, 28, 56 and 84. Viability of blood-fed females was then evaluated for up to 96 h after exposure, and insecticidal efficacy was measured as the survival rate of flies fed on the fluralaner-treated dogs versus that of dogs in the control group. Significance was calculated for the proportion of live fed sand fly counts from treated versus control group dogs.

**Results:**

Comparison of the survival proportions between treated and control groups showed that fluralaner insecticidal efficacy was highly significant in both trials (*P* < 0.001 or *P* < 0.01 in different assessments) through to day 56. In the first trial, efficacy reached 100% on days 1 and 28, and 99.1% on day 56; in the second trial, the insecticidal efficacy was 98.5, 100 and 85.9%, respectively on the same days. On day 84, efficacy was in the range of 53–57% (*P* < 0.05) in the first trial and 0% in the second trial.

**Conclusion:**

A single oral fluralaner administration to dogs under laboratory conditions results in strong and reproducible insecticidal efficacy against *P. perniciosus* for at least 8 weeks.

**Graphical Abstract:**

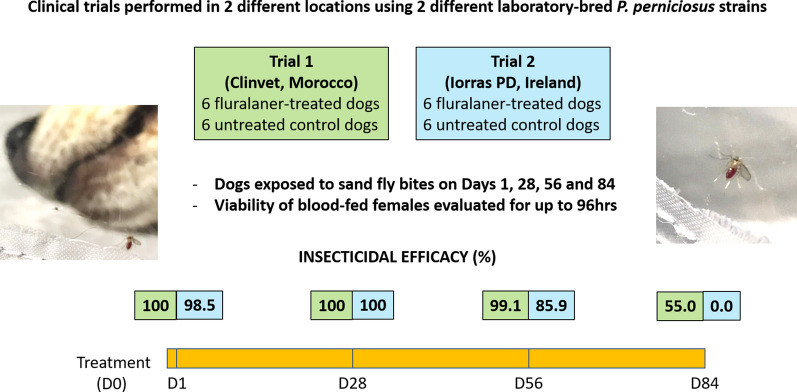

Dogs are the main domestic reservoir of zoonotic visceral leishmaniasis (VL), a protozoal disease caused by *Leishmania infantum* and endemic to all countries of the Mediterranean area, Middle East and Central Asia, as well as several Latin American countries (synonyms: *Leishmania chagasi*; *L. infantum chagasi*) [1, 2]. Canine *Leishmania* infections may result in subclinical disease or an apparently healthy condition, or in clinical disease characterized by increasing severity, sometimes with fatal outcome [3, 4]. The main transmission route of *L. infantum* is through the bite of infected phlebotomine sand flies [5]. Dogs can be infectious to vectors in all stages of infection; however, the duration and severity of leishmaniosis may increase the transmission probability [6]. Starting from about 7 days after an infected blood meal is ingested by a competent vector, infective metacyclic promastigotes develop in the fly foregut and may be introduced into the host’s skin at subsequent blood meals [7]. Sand fly infectiousness is enhanced after a second blood ingestion by reverse metacyclogenesis [8]. *Phlebotomus perniciosus* is the primary vector for *L. infantum* throughout the western Mediterranean basin [9]. *P. perniciosus* is a member of the *Larroussius* subgenus, which includes closely related species also acting as *L. infantum* vectors in endemic areas of the eastern Mediterranean region, such as *Phlebotomus neglectus*, *Phlebotomus perfiliewi* and *Phlebotomus tobbi* [10].

Topical synthetic pyrethroids are recommended as the first-line approach to protect healthy dogs from the feeding of potentially infectious sand flies and also to prevent further parasite spreading via the vector from dogs with leishmaniosis [11, 12]. Isoxazolines are a novel class of systemic insecticides with lethal effects against arthropod vectors [13], and these agents as oral and topically administered formulations are approved veterinary drugs for the protection of dogs and cats against ectoparasites [14, 15]. The comparative efficacy of commercially available isoxazolines was evaluated in dogs against flying insects of medical importance, such as *Triatoma infestans*, a domestic vector of *Trypanosoma cruzi*, the cause of Chagas’ disease [16], and *Phlebotomus papatasi*, the vector of *Leishmania major*, the cause of Old World cutaneous leishmaniasis [17]. In both studies, fluralaner (Bravecto® chewable tablets; MSD Animal Health, Merck & Co., Inc., Kenilworth, NJ, USA) was the most active insecticidal compound based on the level of efficacy and duration of activity. This resulted in the initiation of clinical trials of fluralaner in dogs as an insecticidal treatment targeting phlebotomine sand flies [18], including the New World VL vector sand fly *Lutzomyia longipalpis* [19]. To date, partial data are available from the first clinical trial of fluralaner investigating the onset and peak of insecticidal efficacy against *P. perniciosus* feeding on treated dogs [20]. Here, we present the results from two clinical trials performed in different locations against *P. perniciosus* from different sources, which include the partial data published previously [20].

Two parallel-group, negative-controlled, randomized, blinded clinical trials were performed using purpose-bred beagles at two animal research facilities: Clinvet (Mohammedia, Morocco) and Iorras Product Development (IPD; Glenamoy, Ballina, Ireland). The trials conducted at the two locations followed an equivalent experimental design, with only minimal differences. The challenges employed specific pathogen-free laboratory-bred strains of *P. perniciosus* from two different sources: Istituto Superiore di Sanità (Rome, Italy) and Charles University (Prague, Czech Republic). Specimens aged 2- to 9 days were placed in plastic pots, with each pot having similar age proportions and the number of sand flies in each pot being sufficient for challenging one dog. The insects were shipped by air inside preheated containers which, upon delivery, were stored at 25 ± 2 °C pending challenge, which was performed the following day. At 30 min before challenge, sand flies were released into an empty metal frame and net construction challenge cage (40 × 40 × 58 cm) to acclimatize.

To assess individual attractiveness to the vector under experimental conditions, 14 or 16 beagles, depending on the trial, were sedated with medetomidine hydrochloride at the dose of 0.06 ml/kg body weight, following which they were positioned so that their head was in the challenge cage where it was exposed to bites from an average of 70 live unfed *P. perniciosus* females, together with 5–10 males to promote feeding. After 60 min the dog was moved and the head taken out of the challenge cage; female flies were gently collected through mouth aspiration and macroscopically examined to determine blood engorgement. The dogs were ranked based on pre-treatment counts of blood-fed flies, and two or four dogs with the lowest counts were excluded from the the subsequent respective trial. In both trials, the remaining 12 dogs were randomly allocated to two groups of six dogs each. Dogs in the treated group were administered fluralaner orally on day 0 at the approved commercial dose of 25–56 mg/kg body weight, while dogs in the control group were not treated. On days 1, 28, 56 and 84 after treatment, all dogs were challenged with sand flies in separate rooms maintained at similar temperature and humidity conditions. Following the challenge, all collected blood-fed and unfed female flies were pooled separately in groups of ≤ 10 flies inside plaster-lined plastic pots for viability assessments. The pots were placed in boxes provided with saturated glucose and humidified filter paper and maintained at 25 ± 2 °C. Fly viability was assessed at 6 h, and then at 24-h intervals to a maximum of 96 h following exposure. The proportion of live fed sand flies to the total challenge population was determined.

Insecticidal efficacy was calculated using the proportion of live fed sand fly counts from the treated group versus the control group. The significance of the difference between the proportions was calculated using a linear mixed model that included the study group as a fixed effect and a randomization block as a random effect, with the level of significance set to *α* = 0.05 (two-sided). The model used the Kenward–Rogers adjustment to determine the degrees of freedom of the denominator.

Over 2800 *P. perniciosus* females fed on dogs in the challenge trials (1509 in trial 1 and 1315 in trial 2) and were monitored for mortality over the subsequent 96 h (Table 1). The cumulative number of sand flies that fed on treated versus control dogs did not differ significantly between the two trials, with the ratio being 1.2 in trial 1 and 1.3 in trial 2 (Chi-square test, *P* = 0.19; Chi-square value = 1.73; *df* = 1). No treatment-related adverse events were observed in any dog. Fed *P. perniciosus* survival was used to calculate insecticidal efficacy (Table 2). Significant insecticidal efficacy (41.6%; *P* < 0.05) was observed at the 6 h evaluation after challenge on day 1 in trial 2 (evaluation not done in trial 1), which shows that there was an early onset of activity after treatment. Significant insecticidal efficacy (35.5%; *P* < 0.05),was recorded at 6 h on day 28 in trial 1 (*P* < 0.001), but it was not confirmed in trial 2. From this assessment time point onwards no early insecticidal efficacy was shown. The insecticidal efficacy was similar in the two trials at the 24 h evaluation for the day 1 and day 28 challenges, with 100% insecticidal efficacy on both days in trial 1, and 98.1% and 100% in trial 2. Significant insecticidal efficacy was highest at the 96 h assessment on day 56—with 99.1% insecticidal efficacy in trial 1 and 85.9% in trial 2. On day 84, a significant insecticidal effect was observed at the viability assessments at 48–96 h (52.7–57.2% efficacy) in trial 1, whereas efficacy was not observed in trial 2 at this time point after treatment.

**Table 1:** Cumulative population of blood-fed *Phlebotomus perniciosus* that were collected at the end of challenge and subsequently evaluated for survival following feeding on dogs that were either treated with fluralaner or not treated

Days following treatment	Trial 1 (*n *, blood-fed *P. perniciosus*)			Trial 2 (*n*, blood-fed *P. perniciosus*)		
	Untreated group	Fluralaner-treated group	Total	Untreated group	Fluralaner-treated group	Total
Day 1	64	78	142	134	85	219
Day 28	146	199	345	87	100	187
Day 56	273	303	576	106	190	296
Day 84	206	240	446	241	372	613
Total	689	820	1509	568	747	1315

**Table 2:** Survival of blood-fed *Phlebotomus perniciosus* female sand flies and calculated insecticidal efficacy following feeding on fluralaner treated or non-treated dogs

Days following treatment	Survival assessment time point (h)	Trial 1			Trial 2		
		Mean survival proportion^a^ (%)		Insecticidal efficacy^b^ (%)	Mean survival proportion^a^ (%)		Insecticidal efficacy^b^ (%)
		Untreated group	Fluralaner-treated group		Untreated group	Fluralaner-treated group	
1	6	ND	ND		99.4	58.1	41.6*
	24	89.2	0.0	100***	97.4	1.8	98.1***
	48	76.0	–	–	95.9	1.8	98.1***
	72	67.8	–	–	89.8	1.8	98.0***
	96	61.1	–	–	73.0	1.1	98.5***
28	6	96.9	62.6	35.5*	100	94.4	5.6
	24	96.9	0.0	100***	87.3	0.0	100***
	48	93.5	–	–	61.1	–	–
	72	89.6	–	–	33.9	–	–
	96	80.6	–	–	19.2	–	–
56	6	93.8	89.0	5.1	100	98.1	1.9
	24	81.9	35.8	56.3	100	90.6	9.4
	48	66.8	13.7	79.5**	70.3	16.7	76.3**
	72	58.9	3.4	94.2***	64.1	11.9	81.4**
	96	42.0	0.4	99.1**	61.0	8.6	85.9***
84	6	100	91.7	8.3	100	100	0.0
	24	100	63.6	36.4	94.5	98.2	0.0
	48	92.7	39.7	57.2*	80.8	82.9	0.0
	72	81.7	37.1	54.6*	57.3	71.1	0.0
	96	66.4	31.4	52.7*	46.8	56.6	0.0

ND, Not done

*, **, ***P-value as calculated by the comparison of survival proportions between treated and control groups: Significant at: *< 0.05;,**< 0.01; ***< 0.001

^a^Survival proportion of blood-fed *P. perniciosus* female sand flies per dog (%) = [(live blood-fed females/all blood-fed females) × 100]

^b^Insecticidal efficacy (%) = 100 × [(mean proportion of live fed females in control group − mean proportion of live fed females in fluralaner-treated group)/mean proportion of live fed females in control group]

These results show that fluralaner treatment of dogs provides a high level of insecticidal efficacy against feeding *P. perniciosus* for at least 8 weeks after treatment. On the final assessment at 84 days after treatment there was discordance between the two studies, possibly due to different susceptibility of the sand fly strain to lower plasma fluralaner concentrations [15]. There was an indication that the *P. perniciosus* strain used in trial 2 could be less susceptible because the insecticidal efficacy values observed were consistently slightly lower than those in trial 1 in most determinations performed before day 84 (Table 2). The possibility that dogs in the two trials received different fluralaner doses based on their initial weights was excluded.

With regard to fluralaner activity against different species of phlebotomines, our results show a higher efficacy against *P. perniciosus* than *P. papatasi*, similar to results for pyrethroids (reviewed by [21]). Another fluralaner trial [18] reported 86.5% insecticidal efficacy against *P. papatasi* at 17–31 days post treatment when evaluated 24 h after the exposure of beagles to *P. papatasi*, compared to 100% insecticidal efficacy at post-treatment day 28 when evaluated at 24 h after exposure in both of our trials. A trial evaluating the insecticidal effect of fluralaner against the New World fly *L. longipalpis* [19] recorded an exceptionally long-lasting insecticidal effect with a significant difference between treated and control dogs up to 300 days post treatment. Two possible explanations for the difference between the results of that trial and those of the studies reported here are: a greater intrinsic susceptibility of *L*. *longipalpis* to fluralaner and/or the impact of the extended 5-day period of viability assessment used in [19]. In the *L. longipalpis* study [19], from 90 days post treatment onwards, 100% *L. longipalpis* fly mortality was only observed in the 120 h viability assessments; furthermore, survival data from control groups suggested that blood-feeding under laboratory conditions affects the viability of *L. longipalpis* less than that of *P. perniciosus*. In the latter species, female fly mortality increased sharply after 96 h post blood meal, making a comparison between flies fed on treated or control dogs impossible.

Sand flies can transmit *L. infantum* starting from about 7–10 days after the ingestion of an infected blood meal [7]. The insecticidal efficacy recorded in the present study shows that fluralaner treatment can effectively reduce the risk of *Leishmania* transmission by sand flies that feed on infected dogs for at least 56 days after treatment and potentially longer—as bites are not prevented, however, infected sand flies can potentially transmit *Leishmania* to uninfected treated dogs that they have bitten. Systemic insecticidal effect studies in sand flies should maintain the survival of blood-fed control specimens for as long as possible to detect insecticidal effects that are not initially apparent but still have transmission-blocking potential [20].

Recent published data show that *Leishmania*-infected dogs were significantly more attractive to *P. perniciosus* than uninfected dogs under both laboratory and field conditions [22]*.* A similar behavior was detected when *L longipalpis* was tested [23]. Therefore, treating *Leishmania*-positive dogs with fluralaner in endemic areas with an elevated incidence of human VL may represent a promising tool for reducing the infected *P. perniciosus* population and hence transmission. Community-wide studies in endemic areas would be required to test the impact of this control measure on human and canine disease incidence, similarly to what was done for insecticide-impregnated dog collars [24]. Furthermore, besides providing protection from other vector-borne pathogens, such as those transmitted by fleas and ticks, fluralaner treatment in combination with preventive topical synthetic pyrethroid treatments could provide benefit for all dogs, not just the infected ones, in high-risk *Leishmania* endemic settings. Of note, concurrent application of deltamethrin-impregnated collars was not found contraindicated for the safety of fluralaner-treated dogs [25].

In conclusion, fluralaner treatment of dogs has a high insecticidal effect against *P. perniciosus*-infected feeding sand flies for at least 8 weeks.

## Data Availability

The datasets supporting the conclusions of this article are included within the article.
